# How I wean patients from veno-venous extra-corporeal membrane oxygenation

**DOI:** 10.1186/s13054-019-2592-5

**Published:** 2019-09-18

**Authors:** Francesco Vasques, Federica Romitti, Luciano Gattinoni, Luigi Camporota

**Affiliations:** 1 Department of Adult Critical Care, Guy’s and St Thomas’ NHS Foundation Trust, Health Centre for Human and Applied Physiological Sciences, King’s Health Partners, King’s College London, 6th Floor East Wing, Westminster Bridge Road, London, SE1 7EH UK; 2 Department of Anaesthesiology, Emergency and Intensive Care Medicine, University of Göttingen, Göttingen, Germany

## Introduction

Identifying patients who are ready for weaning and liberation from veno-venous extracorporeal membrane oxygenation (ECMO) is challenging in clinical practice. Compared to the several trials addressing the safety and efficacy of ECMO in severe ARDS [1–4], the body of literature regarding ECMO weaning is remarkably scarce. Therefore, this essential component of the management of patients on ECMO is highly variable and often lacks of a systematic approach [5], analogously to the weaning protocols and spontaneous breathing trials used for liberation from mechanical ventilation [6].

The trajectory from ECMO cannulation to lung recovery and ECMO decannulation consists in the transition from a *phase* in which ECMO is essential to meet the patient’s metabolic needs (i.e. metabolic oxygen consumption and CO_2_ production) to a *phase* in which the native lung function has recovered to satisfy completely the metabolic demands, even if with a degree of ventilatory support considered “safe”. In between these two phases is a continuum of lung healing, during which lung function becomes sufficient to maintain a gas exchange compatible with life, but at the expenses of a high respiratory drive and large swings in transpulmonary pressures. In these conditions, ECMO has the role of maintaining lung protection partially contributing to the patient’s gas exchange [5]. In the effort to track the progress of an individual patient along this imaginary line, it is necessary to measure the relative contribution of the membrane and native lungs in terms of gas exchange, as well as the response of the patient’s respiratory drive and mechanics to the variation in ECMO settings.

We propose a physiology-based assessment protocol, which combines an objective assessment of the native and artificial lung function and quantifies the patient’s response to a standardised weaning trial.

## Patients’ selection

The prerequisites for a weaning assessment is that patients are comfortable, haemodynamically stable and on spontaneous or assisted mechanical ventilation (e.g. CPAP/PS).

In addition, the PaO_2_ after a 100% test (i.e. the systemic arterial PaO_2_ taken after 15 min of FiO_2_ = 1.0 on the mechanical ventilator) > 30 kPa (225 mmHg) and a P0.1 < 5 cmH_2_O.

## Baseline measurements

On a daily assessment of ECMO patients, we routinely measure the following:

- Gas exchange variables, including the 100% test; post-membrane PO_2_, VO_2_ and VCO_2_ of the membrane lung (VCO_2ML_) and VO_2ML_; and VCO_2_ of the natural lung (VCO_2NL_) using volumetric capnometry. This allows calculating the total VCO_2_ (VCO_2tot_ = VCO_2ML_ + VCO_2NL_) and the proportion of VCO_2tot_ eliminated by the natural lung (VCO_2NL_/VCO_2tot_).
- Ventilatory variables, including the negative inspiratory force (NIF), the P0.1 (as estimate of the inspiratory drive) and the ratio of P0.1/NIF, and swings in oesophageal pressure (if available).

**Fig. 1:**
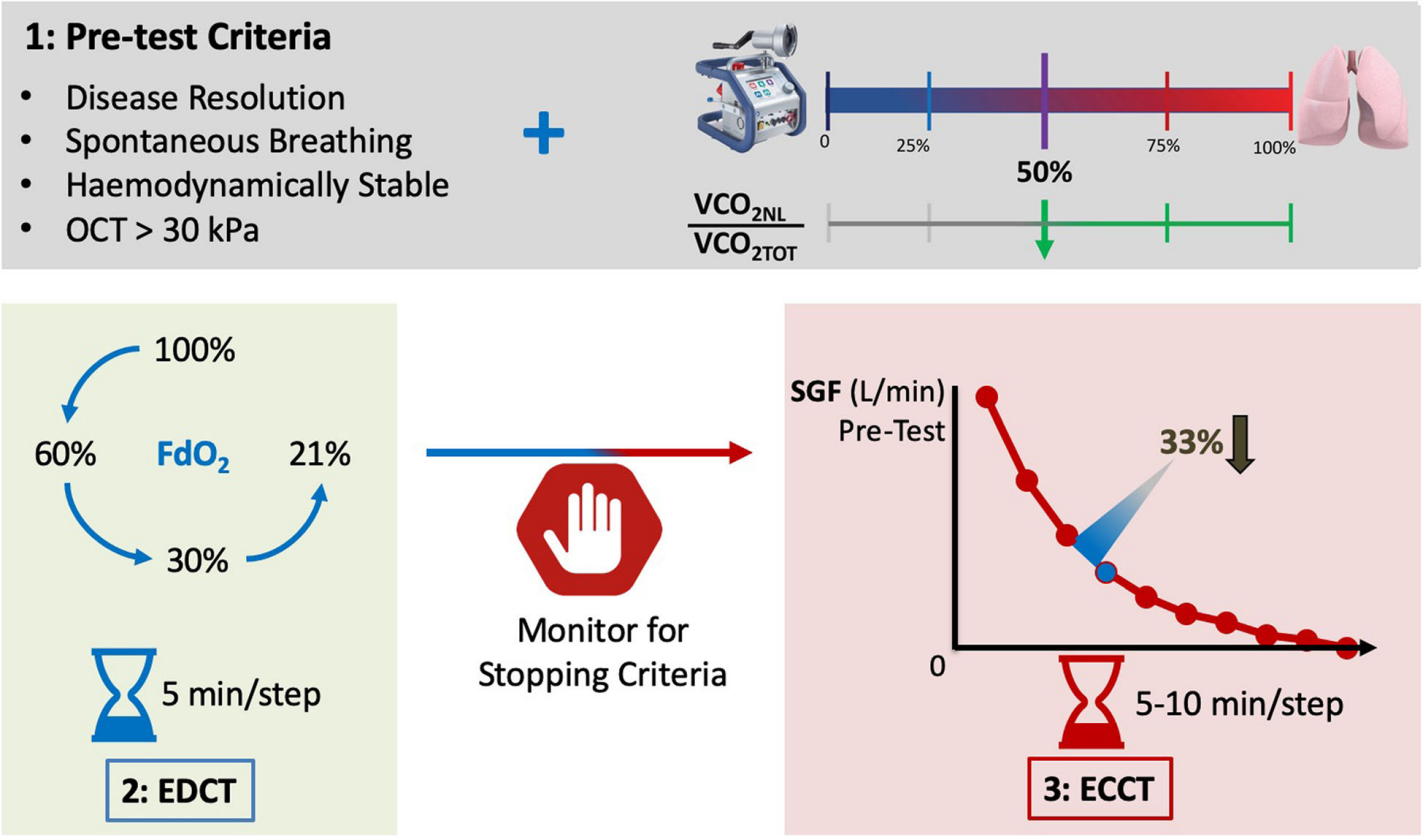
ECMO weaning test. FdO2 = fraction on oxygen in the sweep gas; EDCT = ECMO de-oxy challenge test; ECCT = ECMO CO2 challenge test; OCT = oxygen challenge test (FiO2 100% on the ventilator); VCO2NL = VCO2 natural lung; VCO2ML = VCO2 membrane lung; SGF = sweep gas flow

In our protocol (see Additional file 1 and Fig. 1), we assess separately the patient’s dependence on ECMO—in terms of both oxygenation and decarboxylation, or decarboxylation alone—by conducting two sequential steps. The first step consists in the progressive reduction of the ECMO FdO_2_ (fraction of oxygen in the sweep gas flow), while the second entails the stepwise reduction of the SGF. The ECBF remains unchanged throughout the procedure.

### Step 1: ECMO Deoxy Challenge Test (EDCT)

Before commencing the assessment, the FiO_2_ on the ventilator is increased to 60% in order to prevent the transient hypoxaemia that may happen during ECMO weaning. This frequently observed hypoxaemia is partly explained by the abolition of hypoxic vasoconstriction during ECMO support (due to elevated SvO_2_). The reduction of ECMO VO_2_ will reduce the SvO_2_, which will allow the return of the physiologic hypoxic vasoconstriction. This pulmonary vascular response, however, takes time (minutes), and the slow adaptation can lead to a transiently worsened functional shunt (venous admixture). The role of the EDCT is allow time to restore the physiological hypoxic vasoconstriction and optimise ventilation perfusion matching (V/Q). If V/Q is not restored, the functional dead space due to shunt increases and the ability of the natural lung to eliminate CO_2_ can be impaired.

FdO_2_ is decreased from 100 to 60%, 30% and 21% in 5-min steps. A peripheral oxygen saturation (SpO_2_) > 88% and P0.1 < 10 cmH_2_O will need to be maintained throughout the test. If a patient meets stopping criteria, the test is suspended and the patient is declared “ECMO-dependent”.

If EDCT is successful, FdO_2_ can be kept at 21% and the test can proceed to step 2 (Fig. 1).

### Step 2: ECMO CO2 Challenge Test (ECCT)

During this phase of the weaning trial, the SGF is reduced by 30% every 5–10 min, while measuring the patient’s response (see Additional file 1 and Fig. 1). Weaning failure and test interruption are indicated by SpO_2_ < 88%, RR > 35 bpm and P0.1 > 10 cmH_2_O; VCO_2NL_/VE fall by 20% from baseline; and the negative swings of oesophageal pressure are < 15 cmH_2_O, or if any signs of distress/instability are evident (Fig. 1).

If the patient’s response remains within set limits at 0 SGF, the weaning test is successful, and the clinical team will consider whether to remain off ECMO or reintroduce a variable degree of extracorporeal support pending decannulation.

We believe that a standardised approach to ECMO assessment and weaning is essential to identify patients who are no longer ECMO dependent. In addition, it provides clinicians with a reproducible protocol to ECMO liberation and researchers with a tool to compare duration on ECMO in clinical trials where ECMO duration is an outcome measure.

## Additional file

Additional file 1: Weaning protocol. (DOCX 56 kb)

## Data Availability

Not applicable.
