# Supplementary material for: How I wean patients from veno-venous extra-corporeal membrane oxygenation
Source: Crit Care. 2019 Sep 18;23:316. doi: 10.1186/s13054-019-2592-5 (PMC6749626; doi:10.1186/s13054-019-2592-5)
Supplement: Supplementary file 1 — Weaning protocol. (DOCX 56 kb) [file 13054_2019_2592_MOESM1_ESM.docx]

**ECMO Assessment**

**To be documented in the notes during the ward round by ECMO fellow**

| **ECMO Mechanics:** |  |  |
| --- | --- | --- |
| TMP (mmHg) |  |  |
| Resistance (mmHg/L/min) |  | TMP/ECBF |
| **ECMO gas exchange** |  |  |
|  | **Value** | **Calculation** |
| SGF (L/min): |  |  |
| ECBF (L/min): |  |  |
| Post Oxy PO_2_ (KPa): |  |  |
| VO_2_ML (ml/min) |  | (ctO_2_PostOxy-ctO_2_PreOxy)xECBFx10 |
| VCO_2_ML (ml/min) |  | (ctCO_2(B)_PreOxy- ctCO_2(B)_PostOxy)xECBFx25 |
| VCO_2_ NL (ml/min) |  | Volumetric Capnography |
| VCO_2_tot (ml/min) |  | VCO_2_ML + VCO_2_NL |
| % VCO_2_NL (%) |  | VCO_2_NL/ VCO_2_tot |
| VCO_2_NL/VE (mL/min/L) |  |  |
| P0.1 (cmH_2_O) |  |  |

**Proceed to ECMO weaning test if:**

1. Disease resolution
2. Spontaneous breathing
3. Haemodynamically stable
4. PaO_2_ on 100% test > 30 kPa
5. %VCO_2_NL > 0.5

**ECMO Weaning Test**

ECMO nurse, Consultant or Fellow must be at the bedside throughout the test

| **Step 1: ‘ECMO-Deoxy-Challenge-Test’ (EDCT)** | **Timing** | **Measures/Targets** |
| --- | --- | --- |
|  |  | Measure P0.1 – ensure < 5 with sedation or SGF  RASS -1/-2 |
| Maintain same SGF  Place FiO_2_ to 0.6 | Wait 10 min | SpO_2_ > 88% |
| Decrease FdO_2_ from 1 to 0.6, then 0.3 to 0.21 | 5 min/step^(^^[[1]](#footnote-1))^ | SpO_2_ > 88% |
| **Stop if SpO_2_ < 88% > 5 min** | | |
| If SpO_2_ > 88% - FiO_2_ =0.6 and FdO_2_ = 0.21 |  | Passed ECMO-Deoxy-Test |
| **Step 2: ‘ECMO-CO_2_-Challenge-Test’ (ECCT)** |  |  |
| Decrease SGF by 30% every 10 minutes |  | For each step measure  P0.1;  VCO_2_NL/VE;  VCO_2_NL; ETCO_2_ |
| \| **10.0** \| **9.5** \| **9.0** \| **8.5** \| **8.0** \| **7.5** \| **7.0** \| **6.5** \| **6.0** \| **5.5** \| **5.0** \| **4.5** \| **4.0** \| **3.5** \| **3.0** \| **2.5** \| **2.0** \| **1.5** \| **1.0** \| \| --- \| --- \| --- \| --- \| --- \| --- \| --- \| --- \| --- \| --- \| --- \| --- \| --- \| --- \| --- \| --- \| --- \| --- \| --- \| \| 7 \| 7 \| 6 \| 6.0 \| 5.5 \| 5 \| 5 \| 4.5 \| 4 \| 4 \| 3.5 \| 3 \| 3 \| 2.5 \| 2. \| 2 \| 1.5 \| 1 \| 0.7 \| \| 5 \| 5 \| 4.5 \| 4 \| 3.5 \| 3.5 \| 3.5 \| 3 \| 3 \| 2.5 \| 2.5 \| 2 \| 2.0 \| 1.5 \| 1.5 \| 1 \| 1.0 \| 0.5 \| 0.5 \| \| 3.5 \| 3 \| 3 \| 3 \| 2.5 \| 2.5 \| 2.5 \| 2 \| 2 \| 2 \| 1.5 \| 1.5 \| 1.5 \| 1 \| 1.0 \| 0.5 \| 0.5 \| 0 \| 0 \| \| 2.5 \| 2 \| 2 \| 2.0 \| 2 \| 1.5 \| 1.5 \| 1.5 \| 1.5 \| 1.1 \| 1 \| 1 \| 1.0 \| 0.5 \| 0.5 \| 0 \| 0 \|  \|  \| \| 1.5 \| 1.5 \| 1.5 \| 1.5 \| 1.5 \| 1 \| 1 \| 1 \| 1.0 \| 0.5 \| 0.5 \| 0.5 \| 0.5 \| 0 \| 0 \|  \|  \|  \|  \| \| 1 \| 1 \| 1 \| 1.0 \| 1 \| 0.5 \| 0.5 \| 0.5 \| 0.5 \| 0 \| 0 \| 0 \| 0 \|  \|  \|  \|  \|  \|  \| \| 0.5 \| 0.5 \| 0.5 \| 0.5 \| 0.5 \| 0 \| 0 \| 0 \| 0 \|  \|  \|  \|  \|  \|  \|  \|  \|  \|  \| \| 0 \| 0 \| 0 \| 0. \| 0 \|  \|  \|  \|  \|  \|  \|  \|  \|  \|  \|  \|  \|  \|  \| | | |
| From the baseline SGF (light grey area), reduce the SGF following the relative column | | |
| **Interrupt the test if these criteria are present:**   - **P0.1 > 10** - **RR > 35** - **VCO_2_NL/VE decreases compared to baseline > 20%** - **Obvious signs of distress** | | |
| Complete test | **Return FdO_2_ to 1**  **Return SGF to pre-test value** | |

**List of abbreviations**

| **ECBF** | Extracorporeal Blood Flow |
| --- | --- |
| **FdO_2_** | Device fraction of O2 on the ECMO |
| **FiO_2_** | Inspiratory fraction of O2 on the ventilator |
| **ML** | Membrane Lung (i.e., ECMO) |
| **NL** | Native lung |
| **P0.1** | Pressure in the first 100 ms of inspiration |
| **RR** | Respiratory rate |
| **SGF** | Sweep Gas Flow |
| **VCO_2_NL/VE** | Ventilatory efficiency |
| **VCO_2NL_** | Carbon dioxide elimination by the natural lung |
| **VCO_2ML_** | Carbon dioxide elimination by the membrane lung |

1. As ECMO may reduce or abolish the normal hypoxic vasoconstriction, oxygenation can improve over time once hypoxic vasoconstriction is restored. This may take 20-30 minutes. [↑](#footnote-ref-1)
